# Coping With Hurricane Matthew: Lessons Learned on the Importance of Relationships

**DOI:** 10.1093/geroni/igaa057.318

**Published:** 2020-12-16

**Authors:** Allison Gibson, Ethan Engelhardt, Erin Murphy

**Affiliations:** 1 University of Kentucky, College of Social Work, Lexington, Kentucky, United States; 2 UT Arlington, School of Social Work, Arlington, Texas, United States

## Abstract

The effects of natural disasters are daunting among older populations, especially those with intersecting vulnerable social locations, such as low-income aging racial/ethnic minorities. Yet, there is a paucity of literature on these experiences. The purpose of this study was to explore the experiences of this population before and during the 2016’s Hurricane Matthew. Using a modified grounded theory approach, study participants were recruited through a snowball method and through flyers that were posted throughout affected communities. Semi-structured interviews were used in this study. The analysis focused on the identification of themes. The study aimed to gain knowledge of the experiences of 15 aging minorities with Hurricane Matthew (n=15). The sample was primarily 73.3% females, with a mean age of 69, and of racial/ethnic minorities (53.3% Black and 46.7% Hispanic). All participants self-identified as low-income. The main theme that emerged from this study on how low-income aging minorities cope with natural disasters was the reliance on social capital and the importance of human relationships. The results suggest that in the context of having low-income, aging minority rely on a range of support. Some support came through information and tangible help they received from family, friends, church community, and neighbors. Bridging social capital came from federal and non-profit agencies as well as help received from their employers. Pre-disaster planning should focus on supporting older populations in building social capital. This may be especially beneficial for low-income, aging minority, who may lack adequate financial resources on which to rely.

